# Can compressive thoracic cord lesions cause a pure lower motor neurone syndrome?

**DOI:** 10.1136/practneurol-2018-002016

**Published:** 2018-10-03

**Authors:** Chin Yong Kok, Hoskote Chandrashekar, Christopher Turner, Hadi Manji, Alexander M Rossor

**Affiliations:** 1 MRC Centre for Neuromuscular Diseases, UCL Institute of Neurology and National Hospital for Neurology and Neurosurgery, London, UK; 2 Lysholm Department of Neuroradiology, National Hospital for Neurology and Neurosurgery, London, UK

**Keywords:** neurophysiology, motor, myelopathy, motor neurone disease

## Abstract

Compressive lesions of the spinal cord usually cause a syndrome of upper motor neurone weakness, spasticity and sensory loss below the level of the lesion. It has long been recognised that compressive cervical cord lesions may present as isolated lower motor neurone weakness of the upper limbs, a syndrome termed cervical spondylotic amyotrophy. We describe two patients presenting with isolated lower motor neurone weakness of the lower limbs in association with a compressive cord lesion at T11/12, a condition we have termed thoracic spondylotic amyotrophy.

## Introduction

Compressive spinal cord lesions typically result in a combination of upper motor neurone weakness, spasticity, sensory loss and sphincter disturbance below the level of the lesion. Isolated lower motor neurone weakness affecting the upper limbs due to a compressive cervical cord lesion was first reported by Brain and colleagues^1^ in 1952 and has since been termed cervical spondylotic amyotrophy. Postmortem studies of this condition have shown selective degeneration of anterior horn cells at the level of compression.^1^ We describe two patients presenting with slowly progressive isolated lower motor neurone weakness affecting the lower limbs in association with a compressive spinal cord lesion at T11/12.

### Case 1

A 48-year-old right-handed man, who worked as a fireman, presented with a 2-year history of difficulty climbing the stairs and ‘slapping’ feet. In addition, he reported a 10-year history of low back pain associated with a burning sensation of the feet. Bladder and bowel function was normal. On examination, he had bilaterally large calves and proximal lower limb muscle weakness. There was mild weakness of hip flexion (MRC grade 4/5), moderate weakness of knee extension (MRC grade 3/5) and severe weakness of ankle dorsiflexion (MRC grade 2/5). The upper limb examination was normal. At the time of presentation there were no signs of spasticity and all reflexes were present. The sensory examination was normal.

He had an elevated serum creatine kinase (CK) concentration at 1200 IU/L. Nerve conduction studies showed normal compound muscle and sensory action potentials. Electromyography (EMG) showed evidence of chronic denervation and reinnervation in tibialis anterior and rectus femoris muscles (see table 1). A quadriceps muscle biopsy showed fibre-type grouping and changes most in keeping with neurogenic atrophy. His monozygotic twin was examined and was found to be normal (clinically, radiologically and biochemically).

**Table 1:** A summary of the neurophysiology of cases 1 and 2

	Sural nerve Amplitude (µV)/conduction velocity m/s	Common peroneal nerve to EDB Amplitude (mV)	EMG	
			Acute	Chronic
Case 1	14/55	5.1	None	TA, RF
Case 2	6/32	Absent	IL, VL, TA, Gc	IL, VL, TA, Gc

EDB, extensor digitorum brevis; EMG, electromyography; Gc, gastrocnemius; IL, iliopsoas; RF, rectus femoris; TA, tibialis anterior; VL, vastus lateralis.

**Figure 1:**
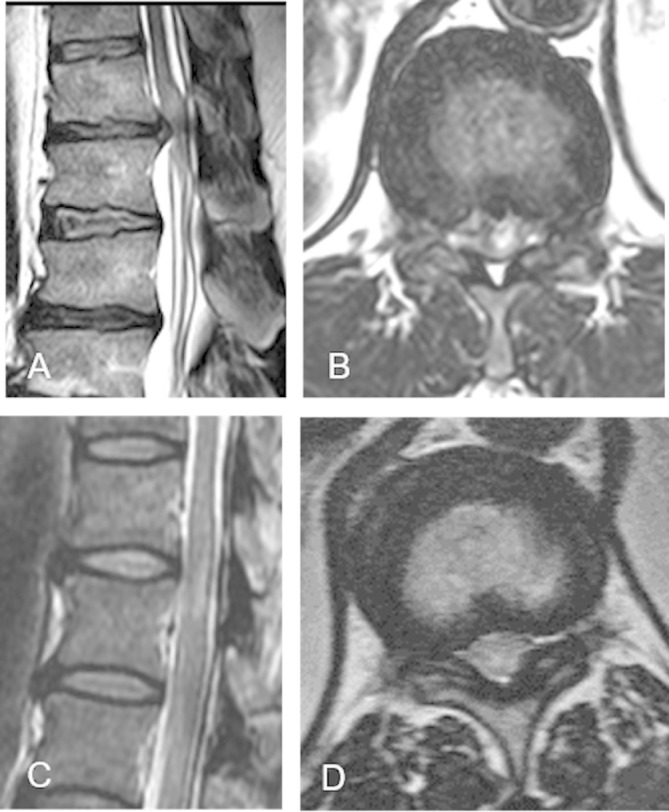
Sagittal T2-weighted MR scan of spine of case 1 (A) and case 2 (C), showing intramedullary signal hyperintensity at T11/12 in case 2 (C). Corresponding axial T2-weighted MRI showing a disc protrusion at T11/T12 level in case 1 (in association with intramedullary signal change) (B) and ligamentum hypertrophy at T11/T12 level in case 2 (D).

**Figure 2:**
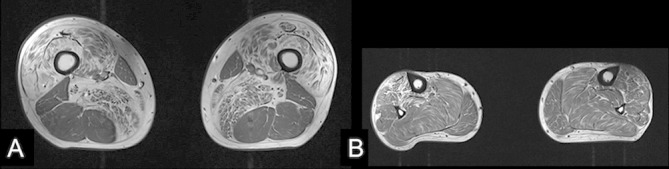
Axial T1-weighted MRI of the thigh (A) and lower leg (B) for case 1 shows fatty infiltration of the quadriceps and adductor muscles (L2, 3 and 4 myotomes; A) and tibialis anterior (L5; B) but sparing of soleus, gastrocnemius and the hamstring muscles (S1).

An MR scan of the whole spine showed a significant disc protrusion at T11/12 resulting in spinal cord compression with associated intramedullary signal change (see figure 1A). An MR scan of the lower limb muscles showed fatty infiltration (a sign of either a primary myopathy or denervation) in the quadriceps, adductors and tibialis anterior but sparing the hamstrings, that is, predominantly L2, L3, L4 and L5 innervated muscles (see figure 2A,B).

The patient continued to progress such that he was only able to walk 50 m unassisted. Following decompression of the T11/12 disc, his exercise tolerance improved and he has since returned to work and can walk unlimited distances. Following surgery he has developed ankle clonus and extensor plantar responses, clinical signs that were absent before surgery. His serum CK concentration has returned to normal.

### Case 2

A 48-year-old man of Caribbean descent presented with a 4-year history of slowly progressive bilateral foot drop progressing to proximal lower limb weakness. His upper limbs were normal and there were no sensory symptoms. More recently he had developed urgency of micturition. His medical history was significant for diabetes and hypertension.

On examination, there was distal muscle wasting of the legs with bilateral foot drop. There were fasciculations in both quadriceps with symmetrical proximal weakness (hip flexion MRC grade 4/5, hip extension 4/5, knee flexion 3/5, knee extension 4/5) and severe distal weakness (ankle dorsiflexion MRC grade 1/5, ankle plantar flexion 2/5). Reflexes were absent in the lower limbs. Joint position sense was impaired up to the ankle and pinprick sensation was reduced up to the knee. The upper limbs were unaffected.

His serum CK concentration was elevated, ranging from 800 to 1000 IU/L. Nerve conduction study showed absent compound muscle action potentials to extensor digitorum brevis and abductor hallucis and normal sensory action potentials (see table 1). Needle EMG showed acute and chronic denervation changes in iliopsoas, vastus medialis, tibialis anterior and medial gastrocnemius muscles (see table 1). A quadriceps muscle biopsy revealed neurogenic changes. An MR scan of spine showed bilateral facet joint hypertrophy and ligamentum flavum hypertrophy with resultant spinal canal stenosis at T11/12 and corresponding intramedullary signal change (see figure 1). The rate of clinical progression has been slow and he has not undergone decompressive surgery.

## Discussion

A pure lower motor neurone syndrome affecting the upper limbs due to degenerative cervical spine disease is well described (cervical spondylotic amyotrophy); however, the existence of a similar syndrome affecting the lower limbs and due to thoracolumbar spinal cord compression is controversial.^2^

Cervical spondylotic amyotrophy is characterised by weakness and wasting of the upper limbs without sensory or lower limb involvement. It can be further classified depending on the muscles involved into a proximal (scapular, deltoid and biceps) and a distal type (triceps forearm and hand). Radiologically, the proximal subtype corresponds to lesions at the C4/5 intervertebral level resulting in damage to anterior horn cells supplying C5/6 nerve root innervated muscles. The distal subtype corresponds to cord lesions at either C5/6 or C6/7 that affect anterior horn cells innervating C7–T1 nerve root innervated muscles.^2^ These anatomical correlations help explain the pathomechanism of disease for the two cases reported in which the cord lesion is at T11/12 but the muscles affected are innervated by nerve roots L2–S1.

The spinal cord enlargement that corresponds to the legs, the lumbosacral enlargement, resides in the vertebral column from approximately T10 to the conus at L1/2. A lesion of the thoracic cord below T10, such as the T11/12 lesions described, can therefore, in theory, damage anterior horn cells innervating the L2–S2 myotomes.

There are two theories for the selective lower motor neurone involvement in cervical spondylotic amyotrophy with a similar pathomechanism likely in our cases. The first is that there is selective damage to ventral nerve roots, a hypothesis supported by a single postmortem study from 1965.^3^ The second (and in our opinion more convincing) theory is that there is selective anterior horn cell damage due to vascular insufficiency. The anterior horn cells lie in the terminal territory of segmental sulcal arteries (branches of the anterior spinal artery) from which they receive their blood supply.^4^ Anterior horn cells are thus more vulnerable to circulatory insufficiency than other cell types that reside within the spinal cord at the same level. In support of this hypothesis, a postmortem study of 145 patients suffering from cardiac arrest or prolonged hypotension revealed ischaemic changes affecting the lumbosacral cord in 50% of patients and predominantly affecting the anterior horn cells.^5^ In the two cases described in this study it is possible that distortion of the sulcal artery at this level results in ischaemia preferentially affecting the most vulnerable anterior horn cells. Furthermore, the sulcal arteries in the lumbar cord may extend up to 1.7 cm longitudinally thereby contributing to the blood supply of motor neurones from overlapping vertebral levels.^4^

The role of surgery in cervical spondylotic amyotrophy is controversial as after an initially progressive onset, the disease usually stabilises and does not progress beyond the affected myotomes.^2^ In our case series, patient 1 had a dramatic response to surgery but was operated on early in the course of his illness at a time when he was deteriorating rapidly. Case 2 has progressed only minimally and has not undergone surgery.

In both cases, the serum CK concentration was significantly elevated suggesting a possible myopathic process. From our experience, a serum CK concentration of up to 2000 IU/L can result from a pure neurogenic process.

Distal hereditary motor neuropathy may cause isolated neurogenic changes in the limbs; however, this diagnosis is unlikely for the following reasons. First, in case 1 the monozygotic twin was unaffected and the patient improved dramatically following decompressive surgery. Second, it is unusual for a distal hereditary motor neuropathy with such severe involvement of the lower limbs to spare the upper limbs.

In summary, we present two cases of a pure lower motor neurone syndrome affecting the lower limbs, associated with a cord lesion at T11/12 that we postulate causes selective damage to motor neurones that reside in the lumbosacral cord at this level. These cases highlight that central cord pathology can give rise to a pure lower motor neurone syndrome. It is unclear whether decompressive surgery can help although the outcome from the first case suggests that it can be if performed early in the disease course.

**Key points**

- Lower thoracic cord compression may cause a lower motor neurone syndrome confined to the lower limbs.
- The lumbar enlargement of the spinal cord corresponds to T11–L1 vertebral levels.
